# What are the most important unanswered research questions in trial retention? A James Lind Alliance Priority Setting Partnership: the PRioRiTy II (Prioritising Retention in Randomised Trials) study

**DOI:** 10.1186/s13063-019-3687-7

**Published:** 2019-10-15

**Authors:** Dan Brunsdon, Linda Biesty, Peter Brocklehurst, Valerie Brueton, Declan Devane, Jim Elliott, Sandra Galvin, Carrol Gamble, Heidi Gardner, Patricia Healy, Kerenza Hood, Joan Jordan, Doris Lanz, Beccy Maeso, Amanda Roberts, Imogen Skene, Irene Soulsby, Derek Stewart, David Torgerson, Shaun Treweek, Caroline Whiting, Sharon Wren, Andrew Worrall, Katie Gillies

**Affiliations:** 1 Health Services Research Unit, University of Aberdeen, Aberdeen, UK; 2 School of Nursing and Midwifery, Evidence Synthesis Ireland, NUI Galway, Galway, Ireland; 3 Birmingham Clinical Trials Unit, University of Birmingham, Birmingham, UK; 4 Department of Adult Nursing, Kings College London, London, UK; 5 Health Research Board-Trials Methodology Research Network, Galway, Ireland; 6 Health Research Authority, National Health Service, London, UK; 7 Department of Biostatistics, University of Liverpool, Liverpool, UK; 8 Centre for Trials Research, Cardiff University, Cardiff, UK; 9 European Patients’ Academy, London, UK; 10 Women’s Health Research Unit, Queen Mary University of London, London, UK; 11 James Lind Alliance, Wessex Institute, University of Southampton, Southampton, UK; 12 Barts Health NHS Trust, London, UK; 13 York Trials Unit, University of York, York, UK; 14 Action on Hearing Loss, British Deaf Association, Deafscotland, Glasgow, UK

**Keywords:** Trials methodology, Retention challenges, Participation in randomised trials, Participant retention, Priority Setting Partnership, James Lind Alliance, Patient and public involvement

## Abstract

**Background:**

One of the top three research priorities for the UK clinical trial community is to address the gap in evidence-based approaches to improving participant retention in randomised trials. Despite this, there is little evidence supporting methods to improve retention. This paper reports the PRioRiTy II project, a Priority Setting Partnership (PSP) that identified and prioritised unanswered questions and uncertainties around trial retention in collaboration with key stakeholders.

**Methods:**

This PSP was conducted in collaboration with the James Lind Alliance, a non-profit making initiative, to support key stakeholders (researchers, patients, and the public) in jointly identifying and agreeing on priority research questions.

There were three stages. (1) First an initial online survey was conducted consisting of six open-ended questions about retention in randomised trials. Responses were coded into thematic groups to create a longlist of questions. The longlist of questions was checked against existing evidence to ensure that they had not been answered by existing research. (2) An interim stage involved a further online survey where stakeholders were asked to select questions of key importance from the longlist. (3) A face-to-face consensus meeting was held, where key stakeholder representatives agreed on an ordered list of 21 unanswered research questions for methods of improving retention in randomised trials.

**Results:**

A total of 456 respondents yielded 2431 answers to six open-ended questions, from which 372 questions specifically about retention were identified. Further analysis included thematically grouping all data items within answers and merging questions in consultation with the Steering Group. This produced 27 questions for further rating during the interim survey. The top 21 questions from the interim online survey were brought to a face-to-face consensus meeting in which key stakeholder representatives prioritised the order. The ‘Top 10’ of these are reported in this paper. The number one ranked question was ’What motivates a participant’s decision to complete a clinical trial?’ The entire list will be available at www.priorityresearch.ie.

**Conclusion:**

The Top 10 list can inform the direction of future research on trial methods and be used by funders to guide projects aiming to address and improve retention in randomised trials.

## Background

Randomised trials are essential for evidence-based health and social care, though many struggle to recruit and retain participants. This poses significant problems for the overall reliability and generalisability of results when recruitment goals are missed or participants are lost to follow-up. One of the top three priorities for research into randomised trials, as set out by the UK clinical trials community, is to address the gap in evidence-based approaches to improving retention in randomised trials [1]. This Priority Setting Partnership (PSP) addresses this subject by investigating unanswered research questions on how to improve retention in randomised trials. Many trial participants drop out before trial completion, sometimes in excess of 20%, and 50% of trials in the UK have a loss to follow-up of more than 11% [2]. As retention within a trial decreases, so too does its credibility and our ability to say with confidence that the results are accurate. This also affects the trial’s ability to contribute towards changes in clinical practice. Without any improvements to retention, trials may be hindered in both their applicability and ability to guide evidence-based care [3].

Despite this, there is currently little evidence other than anecdotal accounts about how to improve retention in randomised trials— as opposed to improvements to recruitment, for which there is substantial evidence. Similarly, the Cochrane review on trial retention at present only has evidence relating to financial incentives to return postal questionnaires and use of second-class versus first-class postage. It also does not include interventions designed specifically to address an individual’s reasons for dropping out of a trial [4]. Current strategies to improve retention in randomised trials could be bolstered by this collaborative PSP, as current guidance is limited to primary care trials only [5] or has been developed without equal patient involvement [6, 7]. To address this, further investigation was necessary with a wider range of stakeholders involved to identify research uncertainties.

These uncertainties about how to retain participants mean that at present sample sizes of trials tend to be increased in anticipation of a loss to follow-up. In addition, many Clinical Trial Units and Chief Investigators use strategies to compensate for missing data with little evidence to support their effectiveness [7]. Around 50% of trials fail to reach their recruitment targets [2], and the average cost of a clinical trial in the UK per participant is around £8500 [8]. Countering loss to follow-up through increases to sample size uses resources that could otherwise be put to better use. Collecting insufficient data to address the aims of a trial reduces our ability to make meaningful use of the generosity of trial participants who enter trials in good faith. It is therefore not only financially prudent to improve trial retention, but important from an ethical perspective in order to reduce research waste and provide better care [9].

For these reasons, there is an urgent need to conduct further research into trial retention. Moreover, this research should be focussed on the areas considered essential to stakeholders who are involved directly. This paper outlines the areas of research that key stakeholders believe should be the focus of future efforts to improve retention in randomised trials. We have defined non-retention as instances in which participants are prematurely ’off-study’, such as withdrawal of consent or loss to follow-up, and therefore outcome data cannot be obtained. This is in line with the Standard Protocol Items: Recommendations for Interventional Trials (SPIRIT) guidelines [10]. This research builds on the methodological framework used in the Prioritising Recruitment in Randomised Trials (PRioRiTy I) project [11], which used a James Lind Alliance (JLA) PSP to identify the top priorities for research into participant recruitment to randomised trials. The JLA method of bringing together relevant stakeholders to decide on research priorities has been used extensively for setting clinical research priorities [12, 13]. However, the application of the JLA method to methodological research is a recent development first utilised in PRioRiTy I [11].

Prioritising Retention in Randomised Trials (PRioRiTy II) viewed the involvement of patient partners in the governance of the project as an integral and essential part of our whole approach. This PSP built and expanded on the PRioRiTy I study by adding more members to the Steering Group. The patient partners included individuals who were new to research methodology as well as some trials methodology-experienced representatives. The decision to supplement the patient partners group with those with less experience of trials methodology research was to ensure that the project continued to ‘talk to’ the wider patient population and link in to networks of said partners who may not have previously been involved in trial methodology research.

## Methods

The methods are described in full in the following sections, but as a brief overview, this PRioRiTy II PSP followed the same overall method as that used during the PRioRiTy I PSP [11]. Key stages included an initial stage that consisted of data collection and analysis to generate a list of unanswered questions. This led onto an interim stage that generated an indicative question list for use in the interim survey. The project then culminated in bringing 21 questions from the indicative list to a final consensus meeting to agree on the top priorities for future research into trial retention. These priorities are uncertainties raised by the stakeholders and judged to be unanswered by existing evidence. All three stages of this project were open to anyone over the age of 18 years who had been involved in randomised trials in the UK and Ireland. For better precision during data collection, seven categories were given as options to describe the role of the stakeholders during the initial survey. For the remainder of the project, we combined stakeholder roles and organised them into the following four groups:

- Patient or member of the public involved in a trial (as a participant or parent/carer of a participant or as a contributor to design/delivery of trial)
- Frontline staff or other staff involved in trial retention (e.g. Research Nurse, Trial Manager, regulatory or oversight role such as Sponsor or Research Director)
- Investigator (e.g. Chief Investigator, Principal Investigator, Co-investigator)
- Trial methodologist

This PSP did not consider uncertainties relating to adherence to trial interventions. The objectives of the PSP were to:

- Bring the public, clinicians, and researchers together to identify unanswered questions around retention in randomised trials
- Agree by consensus on a prioritised list of those unanswered questions which will be used to inform future research.

### Steering Group

We established a Steering Group to oversee the PSP in accordance with JLA guidance and held the first meeting in January 2018. The Steering Group was composed of 24 members: 6 patient partners (3 with experience of trials methodology research and 3 without), 6 frontline staff or other staff involved in trial retention, 5 investigators, 5 trial methodologists, and 2 JLA representatives. Contributors were identified through known personal contacts and key members working in trial retention and invited to join the Steering Group. At the first Steering Group meeting a gap analysis of representation was conducted and efforts made to purposefully fill those gaps through active recruitment, e.g. Twitter adverts for patient partners and direct contacts of known research staff. Membership reflected the range of stakeholders with whom we wished to engage during the PSP. Drawing on members’ expertise and networks, the Steering Group helped identify and recruit stakeholders during each stage. We also held regular meetings to ensure that the work proceeded to agreed timetables and to continue engagement and momentum. The JLA was also represented on the Steering Group to ensure that the process adhered to JLA principles.

### Initial online survey

#### Identification of stakeholders and development of initial survey

Convenience sampling was used to sample survey respondents. Steering Group members, including the patient partners, identified and engaged a wide range of appropriate potential stakeholders through their networks of contacts. The target population mirrored those groups represented by individuals on the Steering Group. Specifically, these were patients or members of the public involved in a trial, frontline staff or other staff involved in trial retention, investigators, and trial methodologists, all of whom needed to be based in the UK or Ireland and be over 18. We developed an eight-question online survey in SurveyMonkey (SurveyMonkey, Palo Alto, CA, USA) to gather uncertainties for our initial stage. This was open for 4 weeks. We also made a paper copy of the survey with pre-paid return envelopes available if required. We set no formal target sample size for the number of responses. The eight questions included six open-ended questions (Appendix 1) that explored the respondent’s views on unanswered questions for trial retention and general comments relating to retention in randomised trials that stakeholders would like to see answered. Based on the experience of the PRioRITy I project and discussion by the project Steering Group, these six open-ended questions were modelled on broad areas of trial retention: why participants stay involved; planning of data collection; processes of collecting data; information provided about data collection; aspects relating to trial staff involved in data collection; any other comments. It was felt that using six questions rather than one generic question may allow broader coverage of all aspects of trial retention. The questions also included an additional two demographic questions about the respondents to help monitor the geographic spread and roles of people responding to the survey. A pilot to test question comprehension and website usability was conducted with a small sample (*n* = 6) of volunteers from within the Health Services Research Unit (HSRU) but included non-academic staff members. We then distributed a weblink to the survey to the four stakeholder groups (described earlier) and also promoted the survey through social media channels and Twitter hashtags. The initial survey was launched in March 2018 and closed in May 2018 (8 weeks of data collection). We also asked respondents if they would consider attending the final consensus meeting. Electronic data was stored on password-protected university computers supported by secure servers. Paper copies of questionnaires were stored in locked tambour filing systems. The electronic and paper data was held in locked offices and only accessible by key personnel.

#### Coding and analysing responses

The initial survey was hosted by the JLA, who provided the Steering Group with regular updates and the compiled answers once completed. We used samples of responses as they were returned to us to identify key themes and questions. This allowed us to generate a representative series of thematic groups efficiently once the survey closed. The JLA collated the survey responses into a single Excel spreadsheet, and we coded the responses using a process of constant comparison analysis [14] into a thematic group where appropriate. We repeated this process of comparison until the range and number of thematic groups truly reflected the whole data set. The determination of thematic groups was an iterative process and generated through discussion amongst the Aberdeen team members, who also conducted the subsequent analysis (DB, HG, KG, and ST). Where an item did not fit into an existing thematic group, we either expanded a thematic group or created a new one. Each stakeholder response could contain numerous items, and multiple themes may have arisen. We therefore sub-divided responses into constituent parts during coding to allow their mapping across different thematic groups. We did not assign responses that were out of scope to a thematic code; rather, we categorised these responses separately for potential future use. All responses that did not refer to a process (such as recruitment) were assumed to be about retention as per the survey questions and hence were considered to be within scope. This process also involved regular group discussion and consultation to ensure consistency in approach and accuracy of the coding.

Once coded, we analysed the data to determine the initial sub-questions and broader main questions present within each theme as well as how often they occurred over the course of 4 weeks. To guide this process, we used word-for-word responses as a framework for developing the sub-questions, which grew iteratively as the data was analysed. We compiled the broader questions from each theme together and conducted a check to ensure that they remained representative of their respective sub-questions. To evaluate reliability, once we connected all coded data items to sub-questions, a 10% sample was selected at random from each team member’s analysis to compare findings. We held group discussions to identify discrepancies and resolve disagreements. We also conducted a check with the questions identified in the initial scoping survey against existing sources of evidence reporting trial retention research. This ensured that questions raised for the interim stage were unanswered by research. The evidence sources used for checking were:

1. The Cochrane review of interventions to improve retention in trials, with the 2012 search updated by members of the Aberdeen team (October 2017) and screened [4]
2. A qualitative synthesis of barriers and facilitators to participant retention in trials [15]
3. A systematic review of non-randomised evaluations of interventions to improve retention in trials, with members of the PRioRiTy II team actively involved as reviewers ([16], with completed review submitted for publication).

Together with the Steering Group, we grouped and merged the longlist of broad questions where appropriate and removed duplicates to create a shortlist of questions in advance of the interim stage. Through consultation with the Steering Group, we discussed and sometimes revised the terminology to improve the clarity of the original meaning of the questions whilst ensuring the items remained true to the voices of respondents.

### Interim priority setting stage

#### Development of the indicative question list and interim survey

In the interim stage, we conducted a ‘back-categorisation’ on the initial stage shortlist in which we asked for feedback and comments from stakeholders who were not involved in the project. These individuals were identified through two processes: (1) email invitation to members of the HSRU (i.e. people who were familiar with trials); (2) invitation to friends and family (who were not familiar with trials) of the Aberdeen team. This process of back-categorisation involved presenting stakeholders with the shortlist of questions from the previous stage and conducting short individual interviews to assess their understanding of the questions. We also asked individuals involved in the back-categorisation process to provide examples of the types of research activities they would expect to see covered by each question. This process ensured that the language used was broad enough to ensure the correct coding of sub-questions under broader indicative questions. For example, questioning around the broad question ‘How could technology be best used in trial follow-up processes?’ included probing stakeholders on what their understanding of ‘technology’ is in relation to trials. Open questions such as ‘From this question, who might you assume would be using technology within trial follow-up processes?’ were used to gather responses to assess whether the question was unintentionally focussing on one specific group of trial stakeholders (e.g. patients). In this example, individuals explained that technology could be used by both the people involved in doing the trial (e.g. research nurses, clinicians) and the people taking part in the trial, so the language of the question was not changed.

The results of the back-categorisation were combined with the earlier responses from the Steering Group to create the list of indicative questions for the interim survey.

For the interim survey, we used SurveyMonkey to ask stakeholders to choose up to 10 of the questions that they believed were the most important. This survey was open for 6 weeks, and we made paper copies of the survey available if required. Invitations to this survey were open to anyone, and not restricted to the participants from the initial survey. As with the initial survey, no formal target sample size was set. However, the number of respondents within each reported group was checked weekly. This allowed us to target groups with lower representation during the ongoing dissemination of the survey.

We distributed the survey link through email, institution websites, blogs, newsletters, and social media. The HSRU at the University of Aberdeen also issued a press release and coordinated promotion alongside the JLA. The interim survey was launched in August 2018 and closed in September 2018 (6 weeks of data collection).

#### Voting and ranking interim survey items

The online survey included a drop-down menu showing the indicative question list from which no more than 10 could be selected. This generated a total score for each question to represent the overall number of times the question was selected. We also used ranked weighted scores to decide which of the interim survey research questions would be taken forward to the final consensus meeting, using the following standard JLA approach as described in the JLA Guidebook [17] (www.jla.nihr.ac.uk/jla-guidebook/).

Each time a question was chosen, we assigned it one point. To ensure equal influence, points for each stakeholder group were tallied separately, generating separate total scores for each group for the questions. Within each of the four stakeholder groups, the scores for each question were arranged in order from highest to lowest. We then gave these a new score according to their position, from 27 for the most popular question down to 1 for the least popular. This resulted in the lowest ranked question receiving the lowest total score, through to the highest ranked question receiving the highest. The list was then ordered by score from highest to lowest and presented to the Steering Group. In cases when the questions had the same total, we ranked them in joint place. This gave the overall interim ranking to the research questions and the rankings for each of the stakeholder groups, whilst minimising bias owing to numbers of responses from each stakeholder group.

### Consensus meeting

The final prioritisation consensus meeting was a one-day event held in Birmingham, UK, in October 2018 to identify and agree on a ‘Top 10’ list of research questions. We brought together representatives from the key stakeholder groups (in roughly equal numbers) to determine the Top 10 list of priorities from the top 21 questions from the interim survey. The consensus meeting followed the standard approach described in the JLA Guidebook, namely using small and whole group discussions in a face-to-face meeting with a particular emphasis on the Top 10 [17] (www.jla.nihr.ac.uk/jla-guidebook/). We remunerated patient participants for their time according to INVOLVE UK guidance, and travel expenses for all attendees were reimbursed. Members of the Aberdeen team planned and organised the event alongside members of the JLA.

The consensus meeting was a full day of plenary and small group discussion, chaired by a JLA Senior Adviser. All attendees were provided with the list of 21 questions in advance of the meeting, to allow time to familiarise themselves with the questions and consider their thoughts on the importance of each one. A JLA facilitator led each of three small groups, which consisted of even representation of the stakeholder groups. The JLA facilitators acted as neutral guides for the process and ensured equal participation in order to minimise authority effects. After an introductory plenary session with the entire group, the three small groups were convened and asked to discuss and prioritise all the listed questions. To support the discussions, individual question cards were used with example quotes from related initial survey responses to provide context. Tri-colour segmented tables were used (red, amber, and green) to represent areas of increasing importance, with red meaning less important and green meaning most important. These initial small groups were then mixed for the second round of discussion and prioritisation to ensure exposure to a range of ideas and eliminate the potential bias of group think. Finally, the small groups all came back together in a plenary session to agree on the final prioritised list.

## Results

### Initial survey

The initial survey was completed by 456 respondents with 454 (99%) answering at least one open-ended feedback question. Only three people requested a paper copy of the initial survey. Completion of the initial survey questions is shown in Table 1.

**Table 1:** Completion of initial survey

Demographic questions	Number	Completed (%)
Consent to participate (yes)	456	100%
Age (range)	452	99%
Respondent’s role in trials	450	99%
Where respondent lives	452	99%
Specific open-ended feedback questions		
Based on your experience, what questions or comments do you have (if any) about why people stay involved in a trial?	454	99%
Based on your experience, what questions or comments do you have (if any) about the planning of study data collection?	415	91%
Based on your experience, what questions or comments do you have (if any) about how trials collect follow-up data from participants?	397	87%
Based on your experience, what questions or comments do you have (if any) about the information people are given about follow-up data collection procedures for a trial?	376	82%
Based on your experience, what questions or comments do you have (if any) about trial staff who are involved in collecting follow-up data from trial participants?	343	75%
Do you have any other questions or comments about how people are encouraged to stay involved in trials?	261	57%

#### Demographic information: initial survey

The most frequently reported role amongst initial respondents was a researcher involved in aspects of trials other than retention (22%). The proportion of respondents within each stakeholder group and the geographical spread are shown in Table 2.

**Table 2:** Initial survey respondent roles

	Number	Percentage
Which one of the following best describes your main role in a randomised trial?		
A researcher involved in aspects of the trial other than retention	96	22
A principal investigator	95	21
A person invited to take part in a trial	70	15
A trial methodologist (someone who specialises in the methods of how trials are designed, run, analysed, and reported)	70	15
A researcher involved in encouraging people to stay involved in trials	65	14
A patient, carer, or public contributor to the design or running of trials	22	5
Other (please describe)	19	4
A parent or carer of a person invited to take part in a trial	10	2
No response	9	2
Total	456	100
Where do you usually live?		
England	281	62
Scotland	61	14
Republic of Ireland	51	11
Wales	33	7
Other	15	3
Northern Ireland	11	2
No response	4	1
Total	456	100

#### Initial stage: collating themes and merging questions

Thematic grouping was used to sort and separate data from the 2431 answers from 456 responses into categories, resulting in 3256 individual coded items. Within this there were 372 specific questions about retention that served as templates to create a series of sub-questions which would later be grouped within and constitute the main questions, depending on how often they appeared within the data. This allowed each coded item to be represented with a question and led to the creation of a longlist of 105 total questions through combining overlapping main and sub-questions. Through review and discussion with the Steering Group, we merged questions from this list where there was substantial overlap to a shortlist of 33 questions to be carried over to the interim stage.

### Interim stage

#### Completion of interim survey

With further consultation and the ‘back-categorisation’ process, we reduced the initial question shortlist to an indicative list of 27 questions to be used in the interim survey. The survey received 886 responses overall. Of these, only 1 was on paper and 16 were not answered fully. The following results therefore report data from 870 responses. Of these, 100% made a selection of up to 10 of the 27 questions, and 864 respondents gave information describing their gender, with 650 (75%) selecting female and 205 (24%) selecting male, 1 using their own term and 8 (1%) preferring not to say.

#### Demographic information

The spread of stakeholder groups and their geographical spread can be seen in Table 3.

**Table 3:** Interim survey respondent roles

	Number	Percentage
Which one of the following best describes your main role in a randomised trial?		
Frontline staff or other staff involved or invested in trial retention (e.g. Research Nurse, Trial Manager, regulatory or oversight role such as Sponsor or Research Director)	403	46
Investigator (e.g. Chief Investigator, Principal Investigator, Co-investigator)	225	26
Patient or public member involved in a trial (as a participant or parent/carer of a participant, or as a contributor to design/delivery of trial)	174	20
Trial methodologist	68	8
Total	870	100
Where do you usually live?		
England	590	68
Scotland	102	12
Wales	72	8
The Republic of Ireland	51	6
Other	30	3
Northern Ireland	19	3
Total	864	100

#### Interim survey ranking progress

Following the standard JLA approach for analysis of the interim survey [17], a ranked weighted score across all stakeholder groups was used to select the top 21 questions to be taken to the consensus meeting. We made this decision as experience from the JLA suggests between 20 and 25 questions are optimal for discussion. We selected 21 rather than 20 because there was a tie between the rankings, where two questions received the same score.

### Consensus meeting

The consensus meeting consisted of 30 stakeholders across the four groups, comprising 12 patients, 9 clinicians, and 9 total researchers and other staff. Some of the patient partners from the Steering Group attended the meeting as observers. Three JLA facilitators, including one who acted as chair, were present at the meeting in addition to 10 observers from the Steering Group. The consensus meeting culminated in a plenary session involving all the stakeholders. This finalised the ordering of the questions, created the Top 10 list shown in Table 4, and ranked the remaining questions (11–21, Appendix 2). All are available online at www.priorityresearch.ie.

**Table 4:** Top 10 research questions prioritised

Overall ranking	Research question
1	What motivates a participant’s decision to complete a clinical trial?
2	How can trials make better use of routine clinical care and/or existing data collection to improve retention?
3	How can trials be designed to minimise burden on staff and participants and how does this affect retention?
4	What are the best ways to encourage trial participants to complete the tasks (e.g. attend follow-up visits, complete questionnaires) required by the trial?
5	How does involvement of patients/the public in planning and running trials improve retention?
6	How could technology be best used in trial follow-up processes?
7	What are the most effective ways of collecting information from participants during a trial to improve retention?
8	How does a participant’s ongoing experience of the trial affect retention?
9	What information should trial teams communicate to potential trial participants to improve trial retention?
10	How should people who run trials plan for retention during their funding application and creation of the trial (protocol development)?

**Fig. 1:**
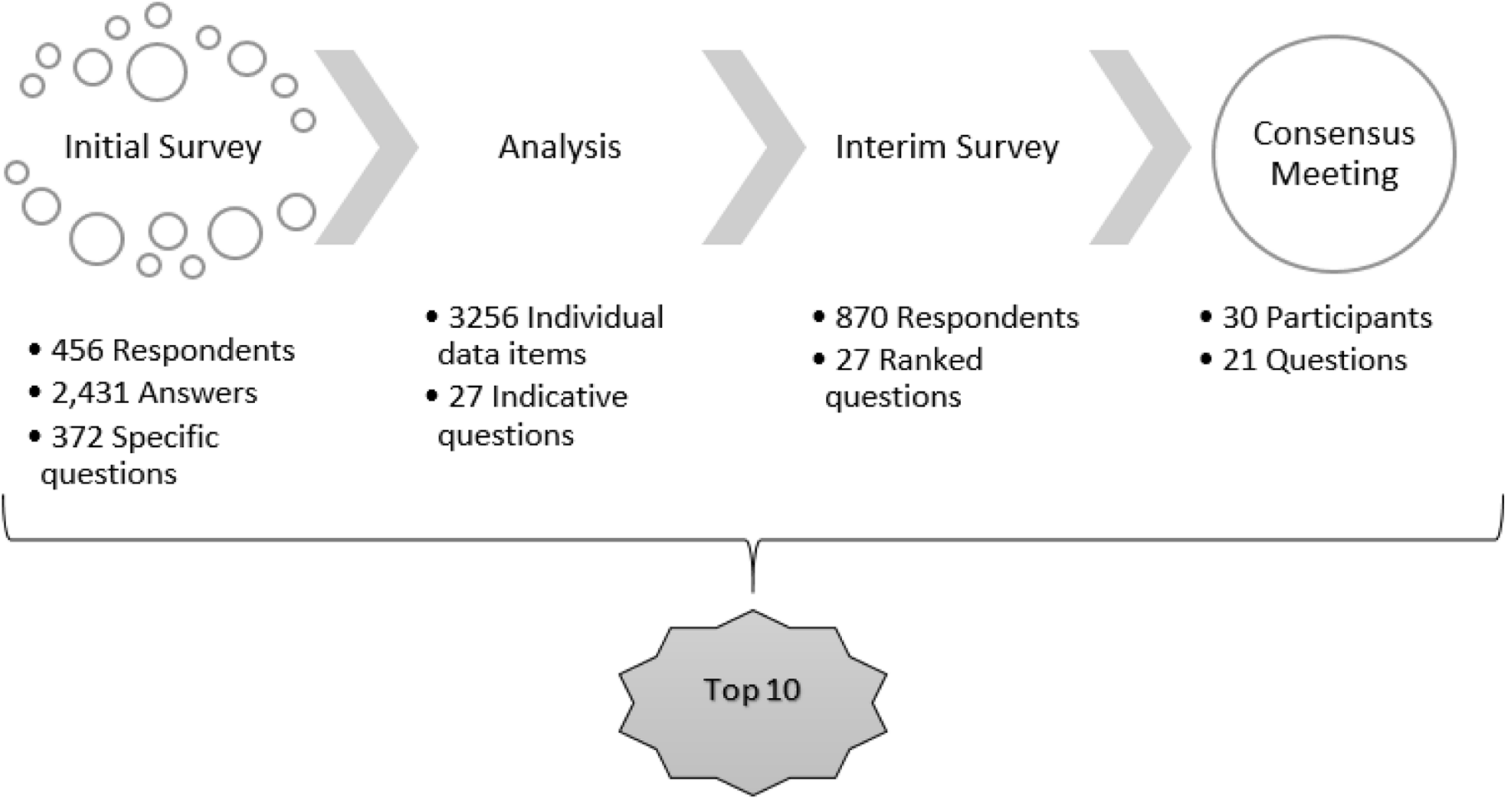
PRioRiTy II priority setting partnership process

The process of this PSP from data collection to the final priority question list is illustrated in Fig. 1.

### Link to PRioRiTy I

During the course of this PSP, there were many similarities between our findings and those from PRioRiTy I. Of our final Top 10 questions generated at the consensus meeting, five share a thematic area with questions within the PRioRiTy I PSP’s top 10 questions for recruitment. These have been highlighted in Table 5 alongside their respective ranking in each PSP.

**Table 5:** Question overlap across PRioRiTy PSPs

	PRioRiTy I ranking	PRioRiTy II ranking	Research question
A	6		What are the key motivators influencing members of the public’s decisions to take part in a randomised trial?
		1	What motivates a participant’s decision to complete a clinical trial?
B	1		How can randomised trials become part of routine care and best utilise current clinical care pathways?
		2	How can trials make better use of routine clinical care and/or existing data collection to improve retention?
C	3		Does patient/public involvement in planning a randomised trial improve recruitment?
		5	How does involvement of patients/the public in planning and running trials improve retention?
D	10		What are the advantages and disadvantages to using technology during the recruitment process?
		6	How could technology be best used in trial follow-up processes?
E	2		What information should trialists communicate to members of the public who are being invited to take part in a randomised trial in order to improve recruitment to the trial?
		9	What information should trial teams communicate to potential trial participants to improve trial retention?

The rows highlighted represent the priorities identified for trial retention

### Availability of the research question list

The entire list of prioritised questions from the PRioRiTy II project will be available online, alongside the results from the PRioRiTy I project into recruitment, on a dedicated website (www.priorityresearch.ie). This was created initially by the Health Research Board-Trials Methodology Research Network Ireland (HRB-TMRN) and expanded to include the results of this PSP. Questions can be viewed by their ranked importance or by thematic category. Teams undertaking research in the areas of these questions are asked to submit details of their work to the HRB-TMRN through the preceding linked website.

## Discussion

Alongside the PRioRiTy I project, we believe the results of this PSP can contribute towards future efforts to reduce research waste and help ensure that the voice of key stakeholders, required to improve retention, is reflected in the direction of future research. Participation was restricted to within the UK and Ireland to ensure that the results were directly applicable in those countries. Therefore, responses from other countries were not used for the final analysis, though international collaboration could help identify common retention issues across randomised trials in different contexts.

As mentioned previously, two linked pieces of work have also developed research agendas within trial retention. The first focussed on developing best practice guidance for the use of retention strategies in primary care trials and built on the findings from the Cochrane review on interventions to improve trial retention [7]. This ‘best practice’ document provides some interesting discussions that may help to add depth to some of the Top 10 questions prioritised in our study. Another prioritisation exercise to identify research priorities for trial retention amongst Chief Investigators identified similar priorities in its Top 10 list [8]. This involved primarily use of routinely collected data (ranked third in their study and second in ours), but they also identified several priorities (e.g. frequency and timing of reminders, time to complete questionnaire, mode of delivery of questionnaire) that could all sit across several of our Top 10 questions. These two studies are complementary, but it is important to note the key difference with our study: the involvement of patients as participants to help identify and set the research priorities. We believe this is a key strength of our work, as recent work exploring the reasons why patients drop out of trials suggests that many of the reasons could be negated through improved patient involvement in the trial design and delivery [15].

### Challenges encountered

An early challenge was the volume of data generated using the six open-ended questions in the initial survey. As we thematically coded and sorted responses during analysis, where a response could contain multiple thematic items and questions within a single answer, we were inevitably faced with the total number of data items being an order of magnitude larger than the total number of initial responses. This type of process of data coding introduces the potential for bias to have been introduced through coder variation, which is a problem for survey research [18]. To mitigate the problem of coder variation, the Aberdeen team worked to ensure a true representation of the data and also conducted the necessary checks and discussions where there was disagreement to ensure the methodological reliability and integrity of the data. We sorted the data so that the connection between individual data items and the final question list was maintained and could be easily viewed and traced. This allows us to demonstrate how initial survey responses were developed into the final priority question list through each stage of this project.

Another challenge faced was the possibility that including examples within a question might influence how the question was interpreted. We sought a balance between ensuring that the questions were clearly explained and trying to avoid unduly influencing their meaning and interpretation. For this reason, and in specific instances, we decided, in discussion with the Steering Group, to give brief examples within questions that might otherwise be hard to understand. However, it was clear from observations of the small group discussions during the consensus meeting that groups in some instances focussed on the examples more than the category the examples represented. In the future, if examples are included within a question, it should be made clear that they are not intended to cover the whole scope of the question, and should not encourage direct answers from the consensus meeting participants. Although, where appropriate, the JLA facilitators and some group participants did point out the difference, it is possible that some people chose to interpret and therefore rank the questions on that basis. In the final ordered question list, the questions ranked in the bottom six positions (16–21) all featured examples. This framing effect is known to be a problem in survey research [19]. As such, this calls into question whether these examples limited the perceived potential scope of the research question and affected stakeholders’ understanding of their comparative importance.

A concern during this project was the potential for one stakeholder group to unduly influence another due to having more extensive knowledge and experience of trial methodology. This could be exacerbated by the difficulties in advertising the project to patients and carers who are unfamiliar with research terminology. At each stage, we acted to ensure that researchers and trial methodologists, who made up a majority of responders during the initial and interim phases, did not overshadow the input of patients and members of the public. This required regular checking of survey response totals to inform our audience targeting, as well as utilising a weighted system of point allocation during the interim survey ranking to provide a balanced perspective of respondent views. We also took steps to ensure that the research remained easily accessible and understandable to all stakeholders during the course of the project. These steps included working closely with the Steering Group during the initial stage to combine similar questions and conducting ‘back-categorisation’ on the terminology of research questions that were generated at the interim stage. We also deliberately omitted titles and job descriptions from name badges at the final consensus meeting to promote equity between the stakeholder groups. Further, the guidance of experienced JLA facilitators promoted a respectful discussion with equal participation. For this entire process the input and contribution of our patient partners was invaluable, as they provided guidance and feedback throughout the PSP. The involvement of patient partners enriched the methods and results of this project.

It is worth noting that the reductionist approach to combining stakeholder sub-groups into four overarching groups may have resulted in some key differences between these smaller sub-groups being missed. For example, perhaps the opinions of patients actively involved in research as partners varied compared to those of patients who had been involved in trials as participants; likewise, Trial Managers’ perspectives could have differed from those of Research Nurses. However, given the overall purpose of the project was to reach consensus across the groups, these specific differences between groups are of less importance than the overall agreement on the research agenda.

During the final consensus meeting, one issue encountered was maintaining the topic of discussion to be focussed on retention rather than branching out onto issues of recruitment, as these two subjects overlap considerably. The experience of the JLA facilitators was indispensable to direct conversation back to the topic and question at hand. This allowed members of the Steering Group and research team to act as impartial observers unless asked to clarify a point by the JLA facilitator, though clarifications were rarely required. Through these combined efforts, we believe we were able to accurately and fairly represent the views of stakeholders, taking due consideration for patient and public contributors, and create an equal opportunity for all to affect the project outcome. Our experiences of communicating and involving members of the public as stakeholders for this meeting are in line with existing research [11, 20–22] in that a flexible approach and quick response were essential.

### Implications

Through our approach, this PSP has successfully identified uncertainties and unanswered research questions on retention in randomised trials. This now provides a platform for future research projects on retention in trials to build upon, and creates a credible methodological approach to the selection of these research topics. This can provide a higher level of certainty for both funders and research organisations that the questions identified in this project are critical to address in any effort to improve retention in trials and reduce research waste [9]. When viewed with PRioRiTy I, we are able to give direction to future research into two of the top three research priorities identified by the UK clinical trials community [1]. Future research to address the third priority, choosing appropriate outcomes to measure, would benefit from a similar approach as reported here such as the Core Outcome Measures in Effectiveness Trials (COMET) Initiative, which uses a similar method of Delphi survey and consensus meeting to develop agreed standardised sets of outcomes for clinical trials, known as ‘core outcome sets’ [23]. Furthermore, by viewing the results of both of these PSPs thematically, we can observe the overlap between uncertainties across retention and recruitment to randomised trials, as well as specific topic areas stakeholders view as important.

## Conclusions

This PRioRiTy II PSP found that the key stakeholders involved in randomised trials such as staff, researchers, and patients/public believe future research on improvements to retention should focus primarily on individual motivation to complete trials, how trials can better use routine clinical care and existing data collection pathways, and how burden to participants can be minimised through trial design. Addressing these concerns is central to any sincere effort to investigate retention within trials as well as more efficiently provide a benefit to patients and others who use our health services. The complete 21-question list will be hosted online at www.priorityresearch.ie and will be further grouped into the thematic comparisons generated by the previous PRioRiTy I PSP on recruitment.

Researchers are encouraged to build proposals addressing the questions raised. We also encourage funders to incorporate these research priorities into their current strategies to address issues with randomised trial retention. We also highly valued having patients and carers as our research partners throughout this project and advocate their contribution as essential for future research into trial retention and trial methodology.

## Data Availability

The data sets generated and/or analysed during the current study are not publicly available due to privacy protections but are available from the corresponding author on reasonable request. The full results of this study are available at www.priorityresearch.ie.

## Appendix 1

**Table 6:** Initial survey question list

Specific open-ended feedback question	
1	Based on your experience, what questions or comments do you have (if any) about why people stay involved in a trial? Motivators might be cash incentives, additional doctor appointments, career benefit, to improve healthcare for themselves and/or others, hope, commitment, etc.
2	Based on your experience, what questions or comments do you have (if any) about the planning of study data collection? Planning trial follow-up includes deciding what should be considered as important to measure, when things should be measured, how they should be measured, where participants should complete follow-up (e.g. at clinic or at home), how they should complete follow-up (e.g. questionnaire), how many people are needed to complete follow-up, what should happen to data for those who don’t complete follow-up, why people don’t complete follow-up, what the impact is on others (e.g. work, dependents, etc.).
3	Based on your experience, what questions or comments do you have (if any) about how trials collect follow-up data from participants? This might include things like who contacts participants, what type of follow-up is appropriate, what types of consequences should be considered (e.g. a clinic visit might require travel), what equipment is needed, how those taking part will know they have to complete follow-up procedures, etc.
4	Based on your experience, what questions or comments do you have (if any) about the information people are given about follow-up data collection procedures for a trial? This might include information from the trial team that is provided to help people decide whether or not to take part in the trial; it might be the questionnaires that are sent to trial participants or a letter with an appointment for a clinic visit. There may also be information sent as a way of helping participants to return the questionnaire or attend the clinic, a prompt or reminder such as a telephone call, a letter, an email, or a text message.
5	Based on your experience, what questions or comments do you have (if any) about trial staff who are involved in collecting follow-up data from trial participants? The trial staff might include a family doctor, a research nurse, a consultant, or other clinicians involved with the trial, or a person employed by a university or other organisation tasked with conducting the research.
6	Do you have any other questions or comments about how people are encouraged to stay involved in trials?

## Appendix 2

**Table 7:** Prioritised list of research questions 11–21

Overall ranking	Research question
11	What aspects of trial recruitment processes could be changed to improve retention?
12	What aspects of trial retention do participants perceive as burdensome, and how can these be addressed?
13	What influence does the relationship between trial staff and participants have on retention?
14	How does a sense of belonging or being part of something amongst trial participants affect retention?
15	What are the best approaches for designing and communicating information about trial retention for trial participants?
16	To what extent (if any) do studies that explore retention procedures before the main trial (i.e. feasibility study) lead to improvements in retention in the main trial?
17	What is the impact of timing, frequency, and duration of follow-up (e.g. questionnaires, clinic appointments) on retention?
18	What strategies (e.g. sending Christmas cards or saying ‘thank you’) make participants feel valued and how do they affect retention?
19	What are the best strategies for using participant incentives (e.g. monetary or non-monetary), and how should they be implemented (e.g. when should they be provided) when collecting information from participants in clinical trials?
20	How does continuity (e.g. seeing/speaking to the same staff) and consistency (e.g. of trial information) affect retention?
21	What behaviours of trial staff (e.g. being friendly) result in improved retention?

## Abbreviations

HRB-TMRN: Health Research Board-Trials Methodology Research Network
HSRU: Health Services Research Unit
JLA: James Lind Alliance
PRioRiTy: Prioritising recruitment/retention in randomised trials
PSP: Priority Setting Partnership
